# Suicide risk of male State patients with antisocial personality traits

**DOI:** 10.4102/sajpsychiatry.v26i0.1543

**Published:** 2020-10-23

**Authors:** Hendrik S. Bosman, Charl Janse van Rensburg, Gian Lippi

**Affiliations:** 1 Department of Psychiatry, Faculty of Health Sciences, School of Medicine, University of Pretoria, Pretoria, South Africa; 2 Biostatistics Unit, South African Medicine Research Council, Pretoria, South Africa

**Keywords:** suicide risk, antisocial personality disorder, forensic psychiatric population, Beck’s Suicide Ideation Scale (BSIS), antisocial personality traits

## Abstract

**Background:**

Suicide mortality rates are higher in people with personality disorders, especially those who have antisocial personality traits. These mortality rates are also higher in people who have committed offences. Antisocial personality traits are very common in populations who have committed offences and in forensic psychiatric patients.

**Aim:**

To determine if male State patients with antisocial personality traits had a higher risk of suicide compared with patients with no antisocial personality traits. We tried to identify other risk factors for attempted suicide in this population.

**Setting:**

Weskoppies Hospital’s Forensic Unit, Pretoria, South Africa.

**Methods:**

Of the 275 male State patients, 37 had antisocial personality traits and were included in the study. Of the remaining State patients, we randomly selected 37 control group participants, who had no antisocial personality traits. For each participant, we completed a data capturing sheet and a Beck’s Suicide Ideation Scale (BSIS). We compared suicide risk and associated factors between study and control group participants.

**Results:**

Study group and control group participants had the same current suicide risk. Overall, 63 participants (85.14%) had no current suicide risk. Of the 11 (14.86%) remaining participants with current suicide risk, 5 had antisocial personality traits. Eighteen had previous suicide attempts, 13 of whom had antisocial personality traits.

**Conclusion:**

State patients with and without antisocial personality traits had similar current suicide risk. Although antisocial personality disorder is an identified risk factor for suicide, it was not the case in this study. Assessment of other risk factors for suicide should be prioritised.

## Introduction

Antisocial personality disorder (ASPD) is associated with early death.^1,2^ Mortality rates because of suicide are also higher in individuals with personality disorders,^3,4^ especially individuals with antisocial personality traits.^5^

These mortality rates are higher in individuals who have committed offences.^6,7^ Antisocial personality traits are very common in populations who have committed offences and in forensic psychiatric patients.^8,9^

The prevalence of personality disorders in the general population is 13.1%, 14.6% amongst women and 13.7% amongst men.^10^ The most prevalent personality disorders are avoidant, paranoid, histrionic and obsessive-compulsive personality disorders. One of the less common personality disorders is ASPD.^10,11^ Suicidal behaviour is most prevalent in individuals with high levels of antisocial traits, borderline traits and narcissistic traits. The mortality rate for offenders with personality disorders is 12 times higher than the general population,^6^ with ASPD or psychopathic traits being a probable risk factor.^11,12,13,14,15^

A previous study has shown higher suicide rates in individuals with psychopathic (antisocial) traits in the correctional services facilities, but not many studies have been conducted regarding suicide rates in the individuals having antisocial personality traits in the forensic psychiatric population in South Africa.^12^ Suicide is a leading cause of mortality in the correctional services and forensic settings, accounting for 6% of deaths. This may be because of a combination of mental illness being over-represented in these settings and the stress of incarceration and arrest.^16,17^ This is especially true for men, who are far more likely to die by suicide, possibly because of more lethal methods used when compared with women.^18,19,20^ Possible reasons for the high prevalence of suicide in individuals with antisocial personality traits could be high negative emotionality, low constraint,^13^ an inability to empathise with others, a distorted self-view, excessive risk-taking behaviour, antagonism characterised by hostility or grandiosity and an increased likelihood to engage in behaviour to manipulate others for personal gain.^15^

Research regarding antisocial personality traits and suicide risk in the South African forensic setting is limited. If individuals with suicidal tendencies can be identified, morbidity and mortality can potentially be reduced. The high prevalence of ASPD amongst prisoners markedly increases the complexity of management and suicide risk.^19,21,22^ We tested if forensic psychiatric patients with antisocial personality traits had a higher risk of suicide compared with patients with no antisocial personality traits in a South African forensic psychiatric setting. This study provides evidence to support the assessment of suicide risk amongst these individuals to prevent suicide-related morbidity and mortality.

## Methods

### Study design

This study was a cross-sectional, comparative and quantitative study. Data were collected during interviews with patients (both inpatients and outpatients), where a specifically designed questionnaire and a Beck’s Suicide Ideation Scale (BSIS) were completed. The questionnaire was completed for patients who already had a diagnosis of ASPD or who had antisocial personality traits (study population), and for patients without the disorder or such traits (control group). The suicide risk and associated factors in the two groups were compared.

### Objectives

The primary objective of the study was to determine if male State patients at Weskoppies Hospital, with antisocial personality traits, had a higher risk for suicide. The secondary objective was to determine if there were other factors associated with an increased risk of suicide in this population.

### Setting

The research was conducted at Weskoppies Hospital, Forensic Unit.

### Study population and sampling strategy

**FIGURE 1:**
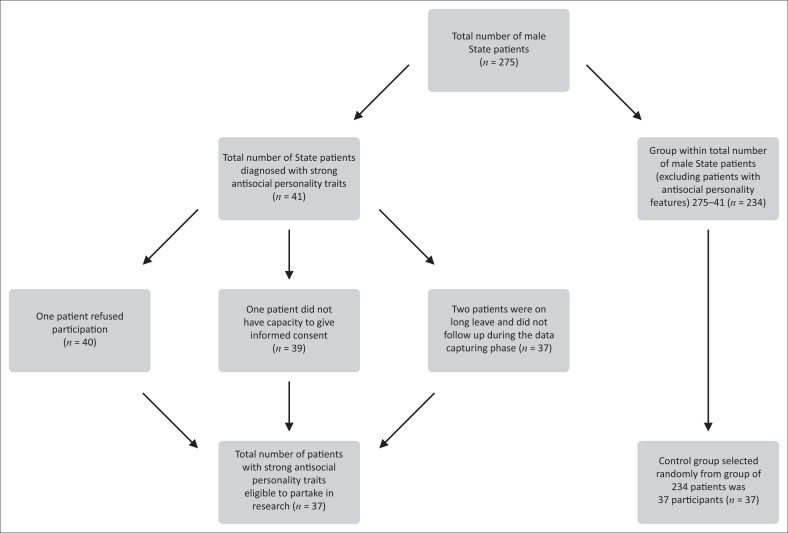
Formation of study population.

We aimed to have a study population and control group of around 45 participants each. The groups eventually included 37 participants each. The formation of the study population is illustrated in Figure 1. Females were not included in the study population as their forensic psychiatric population was too small to participate meaningfully.

The study population consisted of 37 male State patients with antisocial personality traits who are currently known to the Forensic Unit of Weskoppies Hospital, having been referred for care, treatment and rehabilitation in terms of Section 42 of the *Mental Health Care Act*, 17 of 2002.^23^ The control group consisted of 37 male State patients without antisocial personality traits, also known to the Forensic Unit at Weskoppies Hospital. The control group of 37 participants were selected out of a pool of 243 patients by randomly choosing 37 numbers between 1 and 243 uniformly without replacement. All participants were male State patients who gave informed consent.

State patients are forensic psychiatric patients who have committed an offence, but, because of their psychiatric disorder, were found to be unable to follow court proceedings so as to contribute meaningfully to their defence and/or who were incapable of appreciating the wrongfulness of their actions or unable to act in accordance with the appreciation of the wrongfulness of their actions at the time of the alleged offence.^24^

### Data collection

Instruments for data collection consisted of the BSIS and a specifically designed, self-compiled questionnaire, which was completed by a medical professional during an interview with each participant. The questionnaire was compiled in English, and if the participant was not fluent in English, a translator was used.

The questionnaire contained questions about age, race, comorbid psychiatric diagnosis, substance use history, suicidal history, current medication, current ward, offence committed and antisocial personality traits.

### Data analysis

Continuous variables were described using descriptive statistics. Categorical variables were described using frequencies and proportions. Fisher’s exact test was used to test for associations between categorical variables. A 5% level of significance was used. Data were captured using Microsoft Excel and imported into STATA 15, which was used to perform all analyses.^25^

It was recognised that this patient population is a vulnerable population because they receive care, treatment and rehabilitation, not by choice, but as per legal requirement in accordance with the discretion of the treating professionals. Subsequently, we reiterated to all potential participants that they had the right to refuse participation.

All the potential study participants were assessed for the ability to give informed consent to participate in the study. Written or verbal informed consent was used.

Permission to conduct the study was also received from the patients’ district Curator Ad Litem for State patient matters.

Permission was also obtained from the Chief Executive Officer of Weskoppies Hospital to use the clinical files of these patients.

### Ethical consideration

The study was approved by the Faculty of Health Sciences Research Ethics Committee of the University of Pretoria (reference number: 28/2019).

## Results

Of the 74 participants, 63 (85.14%) participants scored zero on the BSIS and 11 (14.86%) participants scored above zero. Of the 11 participants who scored above zero on the BSIS, 5 (6.76%) participants had a moderately elevated score of 1 or 2 out of 18 and 6 (8.11%) participants had higher scores, ranging from 5 to 18 out of 18. Most participants who had low to moderate BSIS scores (0–2 out of 18) did not have antisocial personality traits (*n* = 3). All the participants (*n* = 5) who scored higher on the BSIS (10–18 out of 18) had antisocial personality traits. Overall, the study group and the control group had the same suicide risk, according to the BSIS scores.

Both groups had similar offence profiles (Table 1), but when divided into broad categories, participants with antisocial personality traits had committed more theft offences (*p* = 0.002) compared with participants without antisocial traits (Table 2). When considering psychiatric diagnoses (Table 3), participants with antisocial personality traits were more frequently diagnosed with alcohol use disorder (*p* = 0.081; one-sided Fisher’s exact *p* = 0.040), cannabis induced psychotic disorder (*p* = 0.115; one-sided Fisher’s exact *p* = 0.057) and cannabis use disorder (*p* = 0.162; one-sided Fisher’s exact *p* = 0.081). Other diagnoses were equally distributed between the two groups. When divided into broad categories, both groups had similar prevalence of psychotic disorders, substance use disorders, mood disorders and impaired cognition (Table 4).

**TABLE 1:** Prevalence of the presence or absence of antisocial personality traits in patients of the two groups according to offence committed.

Antisocial personality traits	No antisocial personality traits
Robbery (*p* = 0.054)	Attempted rape (*p* = 0.493)
Housebreaking (*p* = 0.240)	Murder (*p* = 1.000)
Malicious damage to property (*p* = 0.493)	Attempted murder (*p* = 1.000)
Theft (*p* = 0.493)	Rape (*p* = 1.000)
Assault with intent to do grievous bodily harm (*p* = 0.754)	Rape of a minor (*p* = 1.000)
Car theft or attempted car theft (*p* = 1.000)	Sexual assault (*p* = 1.000)
Indecent exposure (*p* = 1.000)	Indecent assault (*p* = 1.000)
Fraud (*p* = 1.000)	Fraud (*p* = 1.000)
-	Possession of firearm (*p* = 1.000)

**TABLE 2:** Prevalence of groups of offences amongst State patients with and without antisocial personality traits.

Offence category	Antisocial personality traits		No antisocial personality traits		*p*
	*n*	%	*n*	%	
Theft offences	9	24.32	0	00.00	0.002
Sexual offences	8	21.62	14	37.84	0.203
Violent offences	20	54.05	21	56.76	0.858

**TABLE 3:** Prevalence of different diagnoses in State patients with and without antisocial personality disorders.

Antisocial personality traits	No antisocial personality traits
Alcohol use disorder (*p* = 0.081; one-sided Fisher’s exact *p* = 0.040)	Schizophrenia (*p* = 0.100)
Cannabis-induced psychotic disorder (*p* = 0.115; one-sided Fisher’s exact *p* = 0.057)	Intellectual disability (*p* = 0.345)
Cannabis use disorder (*p* = 0.162; one-sided Fisher’s exact *p* = 0.081)	Major neurocognitive disorder (*p* = 1.000)
Personality disorder other than antisocial personality disorder (*p* = 0.240)	Mild neurocognitive disorder (*p* = 1.000)
Bipolar disorder (*p* = 0.493)	-
Attention deficit hyperactivity disorder (*p* = 0.493)	-
Stimulant use disorder (*p* = 0.615; one-sided Fisher’s exact *p* = 0.307)	-
Unspecified schizophrenia spectrum and other psychotic disorder (*p* = 1.000)	-
Schizoaffective disorder (*p* = 1.000)	-
Depressive disorder not otherwise specified (*p* = 1.000).	-
Mild neurocognitive disorders (*p* = 1.000)	-

**TABLE 4:** Prevalence of groups of diagnoses among forensic State patients with and without antisocial personality disorders.

Diagnostic group	Antisocial personality traits		No antisocial personality traits		*p*-value
	*n*	%	*n*	%	
Substance use disorders	23	62.16	14	37.84	0.133
Impaired cognition	5	33.33	10	66.67	0.247
Mood disorders	4	80.00	1	20.00	0.358
Psychotic disorder	28	46.67	32	53.33	0.374

In terms of the number of charges per participant, 30 (81.08%) participants with antisocial personality traits and 36 (97.03%) participants without antisocial personality traits had been charged with a single offence. Six (16.22%) participants with antisocial personality traits and 1 (2.70%) participant without antisocial personality traits had been charged with two offences. One (2.70%) participant with antisocial personality traits had been charged with three offences, whilst none of the participants without antisocial personality traits had been charged with three offences.

Regarding substance use disorders and BSIS scores, 35 (47.30%) participants who scored low (< 5) on the BSIS had a substance use disorder, compared with 33 (44.60%) who did not. Out of the 6 participants who scored high (> 5) on the BSIS, 2 (5.41%) had a substance use disorder and 4 (10.82%) did not (*p* = 0.674). Of the participants who had previously attempted suicide, half (9) had a substance use disorder.

When comparing previous suicide attempts with current suicide risk (BSIS score), 15 (83.33%) participants who had previous suicide attempts scored low (< 5) on the BSIS, compared with the 3 (16.67%) participants who scored high (> 5) (*p* = 0.150).When comparing previous suicide attempts between the two groups, 13 (35.14%) participants with antisocial personality traits and 5 (13.51%) participants without antisocial personality traits had previously attempted suicide.

Seven (38.89%) participants who had previous suicide attempts did not have a psychotic disorder diagnosis, compared with 11 (61.11%) participants who had a psychotic disorder (*p* = 0.120).

Three (16.67%) participants who had a history of previous suicide attempts had a mood disorder, compared with 15 (83.33%) participants who did not (*p* = 1.000).

## Discussion

We compared the suicide risk of State patients, with and without antisocial personality traits. Overall, all the patients had the same suicide risk. This is contrary to previous research stating that the risk of suicide is higher in general psychiatric patients with antisocial personality disorders.^5,11,15^ It is possible that we did not detect any differences because our study sample was too limited. Our study sample included patients who had been stabilised in the forensic unit, and there is a possibility that they may have been dishonest because of the nature of their personality structure or fear of being moved to a more restrictive environment. Patients in our study were also being treated and may have had a lower risk of immediate suicide.

In our study, participants with antisocial personality traits were more likely to have previously attempted suicide. Previous suicide attempts are an established risk factor for suicide,^26,27,28,29^ even though previous suicide attempts were more prevalent amongst participants with low BSIS scores (< 5) in our study.

Even though having antisocial personality traits was not associated with increased suicide risk in this study, its association with an increased number of previous suicide attempts makes it a valid variable to consider when assessing suicide risk amongst psychiatric patients.

The participants who scored high (> 5) on the BSIS and had high suicide risk, consisted mostly of participants with antisocial personality disorder. This is consistent with previous research showing individuals with antisocial personality traits having higher suicide risk, a finding which was not replicated in our study. We used the BSIS to assess immediate suicide risk. Previous studies have suggested that the BSIS is useful for continuously evaluating suicide ideation and that the scale should be used repeatedly to identify patients at high risk of suicide.^30^ We did not evaluate suicide risk at the time of offence or the time of admission, and it is likely that assessing suicide risk at specific points in time would reveal differences.

State patients in our study were more likely to present with substance related diagnoses and mood disorders as comorbidities if they had antisocial personality traits. This is consistent with previous research which proposes high comorbidity between ASPDs, substance use disorders and mood disorders.^31,32,33,34^

In our study, participants with ASPD committed more theft-related and less sex- and violence-related crimes when compared with participants without antisocial personality disorder. Previous research that describes a high incidence of violent crimes in individuals with ASPD was not conducted in a South African forensic psychiatric population.^35,36,37^

### Limitations

The study has some limitations. The participants in the study were all male, and the findings may not apply to female patients. The study was conducted at one setting, and therefore, the generalisability of the results is limited. Antisocial personality traits were diagnosed by previous treating healthcare professionals. We did not use a validated scale or tool for diagnostic purposes. We only used the BSIS to assess suicide risk. Language differences between the researchers and the participants might have influenced the gathering of data. Only 11 participants had a score on the BSIS above zero, shrinking our effective population size. The duration of illness and treatment may also have influenced severity and suicide risk.

### Strengths

Despite few patients scoring above zero on the BSIS, the total size of the study population and the control group was sufficient. There is, to our knowledge, no prior research conducted on this topic in this specific patient population in South Africa.

## Conclusion

Our results are contrary to previous research that showed a higher risk of suicide in individuals with ASPD in the general psychiatric population. All State patients in our study had the same suicide risk. Our findings suggest that other well-known variables associated with suicide, such as previous suicide attempts and comorbid substance use disorders, should remain the focus of suicide risk assessment in the forensic psychiatric patient population. If the suicide risk of State patients can be accurately assessed, it can help to provide improved care, treatment and rehabilitation for these patients.
